# Integration of Bioelectronics and Bioinformatics: Future Direction of Bioengineering Research

**DOI:** 10.1007/s40846-016-0185-1

**Published:** 2016-12-10

**Authors:** Shao-Hung Chan, Shuenn-Yuh Lee, Qiang Fang, Huimin Ma

**Affiliations:** 1 Electrical Engineering Department, National Cheng Kung University, Tainan, 70101 Taiwan; 2 School of Electrical and Computer Engineering, Royal Melbourne Institute of Technology University, Melbourne, VIC 3000 Australia; 3 Department of Electronic Engineering, Tsinghua University, Beijing, 100084 China

**Keywords:** Bioelectronics, Bioinformatics, Biological engineering, Healthcare, Implantable, Wearable

## Abstract

This special issue of the *Journal of Medical and Biological Engineering* highlights the field of advanced bioelectronics and bioinformatics. Several papers were considered for this special issue, including those on bioelectronics in wearable and implantable medical devices, such as sensors, and bioinformatics in healthcare, brain cognition, and various neural pathologies. Many investigators contributed original research articles to this issue, demonstrating emerging research fields. More than 20 papers were accepted for publication after a high-quality critical review was conducted, and 14 papers were selected for this special issue. This special issue on bioelectronics and bioinformatics attracted a substantial number of full-paper submissions from many countries. We appreciate the numerous volunteers who helped review the manuscripts. This paper provides a brief review of issues regarding bioelectronics and bioinformatics devices.

## Introduction

Medical science and biology is a crucial research topic. A constant goal of human civilization is to fight and avoid diseases. Engineering has driven the pace of progress in medicine by providing various methods for detecting, collecting, and analyzing signals from the human body to predict and prevent the occurrence of diseases [1]. The proper utilization of information obtained from the human body can enhance life quality. Thus, the advancement of technologies is closely related to the improvement of human health and life quality.

The enormous volume of information collected from the human body requires detailed analysis. The quality of bioinstruments has been improved via numerous techniques. Hardware, such as filters and amplifiers, is widely used to eliminate noise and extract critical physiological signals with high accuracy. Additionally, the availability of wearable devices has gradually increased. Software-based support vector machine (SVM) or other machine-learning programs classify data, analyze trends, and predict consequences when new information is added. With novel algorithms and advanced manufacturing techniques, medical devices have become increasingly portable, functional, and non-invasive.

This special issue includes 15 extended and peer-reviewed papers that were initially presented in the 4th International Symposium on Bioelectronics and Bioinformatics (ISBB2016). These papers cover a wide range of topics, including biomedical signal and image processing, biometric identification, bioinformatics and biomedical signal processing, brain research and brain computer interfaces, computer-aided diagnosis and machine intelligence, healthcare information systems, implantable medical electronics and VLSI IC design for biomedical application, bioinstrumentation, biomechanics, and prosthesis research. This review article provides the basic concept of bioelectronics devices and issue in bioinformatics. Through the integration of bioelectronics and bioinformatics, the future of bioengineering is undoubtedly without limits.

## Bioelectronics Devices

Physiological signals can be accurately detected from human bodies and processed with the aid of electrical devices. Diagnosis, monitoring, therapy, and advanced healthcare treatments require sophisticated engineering design and problem-solving skills [2]. This trend dates back to the early twentieth century when discoveries in basic sciences, such as chemistry, physiology, and pharmacology, gradually emerged. In 1903, William Einthoven invented the first electrocardiograph, which measures the electrical activity of the heart. This invention initiated a new age for cardiovascular medicine and techniques in electrical measurement [3].

One of the components of a bioelectrical device is the biosensor, which transforms physical phenomena into electrical signals [4]. Biosensors are used to detect physical or chemical responses caused by biological behavior [5]. A biosensor is composed of three parts, namely, a recognition element, which is used to sense targets; a transducer, which converts the signals of chemical reactions into analog electrical signals; and an acquisition system, which shapes or digitalizes the signal for analysis and facilitates easy storage [6].

An artificial retina, which contains an implantable image sensor, is a recent application of biosensors. Eye diseases, such as retinitis pigmentosa and aged macular degeneration, may cause photoreceptor cells to lose function; despite these diseases, most inner retinal neurons, such as ganglion cells, remain histologically intact [7]. Therefore, we could create retinal prosthesis vision through stimulating the remaining retinal cells. CMOS technology has been applied for the manufacturing of photo sensors. The CMOS process provides flexibility when matching the curvature of the eyeball for better interconnection of electrodes and external lead wires, micro photodiode array [8], or log sensor [9].

Started in 1967 by Updike and Hicks [10], research on biosensors still has considerable room to grow. Suitable and precise amplifiers and filters are integrated into various sensors. The type of components should be well chosen to enhance the main signal and suppress white noise and achieve high resolution. In this issue, a slow-fast comb filter with feedback is applied to a heart-rate-monitoring device using the photoplethysmogram signal to alleviate motion artifacts [11]. The details of this approach and other interesting research papers on this topic can be found in this special issue.

## Issues in Bioinformatics

Bioinformatics is defined as the process of understanding and organizing biological data by applying computer and programming tools. According to Luscombe et al. [12], bioinformatics is the integration of computer science, statistics, and mathematics. Given that biological data is produced at a phenomenal rate [12], scientists heavily rely on computer calculations while conducting research. Bioinformatics has expanded with the emergence of large databases and ever-increasing processing speeds.

Bioinformatics has been traditionally used in molecular biology, especially in dealing with data related to genomics. Bioinformatics was first introduced in genetic engineering. A DNA sequence determines a protein sequence, which determines protein structure [13]. Raw DNA sequences, protein sequences, macromolecular structures, genomes, and gene expressions contain a large amount of information [12]. The Nucleotide Sequence Database of the European Molecular Biology Laboratory (EMBL) increased from 58.7 million entries in Release 84 (September 2005) to 80.5 million entries in Release 88 (September 2006); this database contains information on over 260,000 organisms [14]. Given its size, this database requires an algorithm that can find data quickly and compare entries. Given that conventional biochemistry sequencing methods can only solve about 1000 base pairs, other algorithms that require powerful computer capability, such as shotgun sequencing, have been introduced [15]. Sequences dynamically change with time, which results in the need for comparison methods. Improvement of molecular biology technology facilitates the discovery of information. With the aid of bio-information, such as distance between atomic coordinates, proteins can be compared either through their sequences or 3D structures; this process reveals independent solutions of crystal structure between identical proteins or distinguishes homologous structures among non-identical proteins [16]. Genomics is related to evolution (phylogeny). Non-long terminal repeat (non-LTR) retrotransposons, which are both short and long interspersed nuclear elements (SINES and LINES), are used to derive phylogenetic relationships [17].

The complexity of computational techniques and methods is far beyond human thought. Machine learning, data mining, text analysis, and data integration have been developed for analyzing protein and RNA structure, protein function, and proteomics [15]. Supervised learning models, such as SVM, have been applied to data classification and regression analysis. If the database have already been classified into training vectors, some of machine learning methods can be used to predict which set a newly-added data [18, 19]. Such a method can be used in multi-voxel pattern analysis (MVPA), which focuses on the analysis and comparison of distributed patterns of activity [20]. This approach can identify differences between conditions with high sensitivity, thereby recursively eliminating irrelevant voxels and estimating informative spatial patterns. This approach is advantageous for neuroimaging and multi-voxel pattern detection of brain activation, which provides information on a subject’s perceptual or cognitive state [20]. Software libraries support various SVM models. LIBSVM, a library for SVM, was developed by the Department of Computer Science at National Taiwan University in 2000. In 2011, the package was updated to aid formulations, which include C-support vector classification, V-support vector classification, one-class SVM, and ξ-support vector regression [21]. SVM is applied in studies in this special issue to monitor solid food intake by recognizing short-period swallowing apnea using a hidden Markov model (HMM) [22]. SVM was also used to identify schizophrenia patients by classifying EEG alpha band power signals [23] and to recognize intraepithelial papillary capillary loops (IPCLs) by combining a convolutional neural network (CNN) and SVM [24].

## Conclusion

Hardware devices allow precise measurement of physiological signals, and software programs allow robust data analysis. Biotechnology has achieved considerable progress in recent years. An increasing number of detailed signals are being detected. In the future, bioelectronics and bioinformatics will continue to improve.

We close this review by thanking the authors for their insightful papers, the reviewers for their detailed comments and constructive suggestions, and the editors for their tremendous support and guidance throughout the whole process.
